# Homocysteine-lowering gene therapy rescues signaling pathways in brain of mice with intermediate hyperhomocysteinemia

**DOI:** 10.1016/j.redox.2018.08.015

**Published:** 2018-08-25

**Authors:** Vanessa Baloula, Marta Fructuoso, Nadim Kassis, Dalale Gueddouri, Jean-Louis Paul, Nathalie Janel

**Affiliations:** a Université Paris Diderot, Sorbonne Paris Cité, Biologie Fonctionnelle et Adaptative (BFA), UMR 8251, F-75205 Paris, France; b Cellular & Systems Neurobiology, Systems Biologyl Program, Centre for Genomic Regulation (CRG), The Barcelona Institute of Science and Technology, Dr. Aiguader 88, 08003 Barcelona, Spain; c AP-HP, Hôpital Européen Georges Pompidou, Service de Biochimie, 75015 Paris, France

**Keywords:** AD, Alzheimer's disease, AAV, adeno-associated viral, BDNF, brain-derived neurotrophic factor, CBS, Cystathionine beta-synthase, hcy, homocysteine, hhcy, hyperhomocysteinemia, HPLC, high-performance liquid chromatography, NMDA, N-methyl-D-aspartate, NMDAR, NMDA receptor, PI3K, Phosphatidylinositol 3-kinase, PP2A, Protein phosphatase 2A, RTK, receptor tyrosine kinases, Hyperhomocysteinemia, DYRK1A, AAV, Brain, RTK pathway, NFkB pathway

## Abstract

Hyperhomocysteinemia due to cystathionine beta synthase (CBS) deficiency is associated with diverse cognitive dysfunction. Considering the role of the serine/threonine kinase DYRK1A, not only in developmental defects with life-long structural and functional consequences, but also in multiple neurodegenerative diseases, its protein expression and kinase activity has been analyzed in brain of heterozygous CBS deficient mice and found to be increased. We previously demonstrated that specific liver treatment with an adenovirus expressing *Dyrk1A* normalizes hepatic DYRK1A level and decreases hyperhomocysteinemia in mice with moderate to intermediate hyperhomocysteinemia. We here use a hepatocyte-specific recombinant adeno-associated viral (AAV) serotype 8-mediated DYRK1A gene therapy (AAV2/8-DYRK1A) to analyze the effect of hepatic *Dyrk1A* gene transfer on some altered molecular mechanisms in brain of mice with intermediate hyperhomocysteinemia. Our selective hepatic treatment alleviates altered DYRK1A protein level and signaling pathways in brain of mice, the MAPK/ERK and PI3K/Akt pathways initiated by receptor tyrosine kinase, the BDNF dependent TrkB pathway, and NFkB pathway. These results demonstrate the positive effect of AAV2/8-DYRK1A gene transfer on neuropathological and inflammatory processes in brain of mice with intermediate hyperhomocysteinemia.

## Highlights

• Plasma homocysteine level is decreased after AAV2/8-DYRK1A gene transfer.
• A positive correlation is found between plasma homocysteine and brain DYRK1A protein levels.
• AAV2/8-DYRK1A gene transfer rescues RTK-mediated signaling pathways.
• AAV2/8-DYRK1A gene transfer increases brain BDNF level.
• AAV2/8-DYRK1A gene transfer rescues NFkB signaling pathway.

## Graphical abstract

**Figure:**
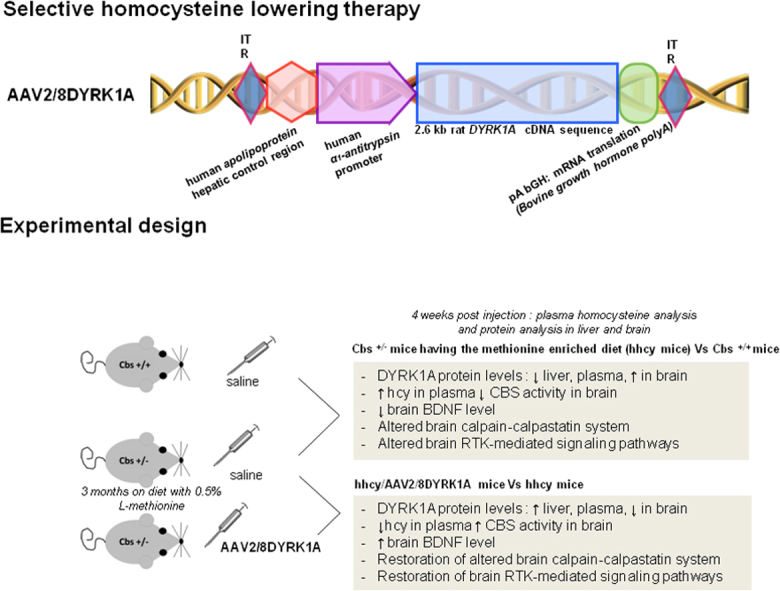

## 1 Introduction

Cystathionine beta-synthase (CBS) deficiency is an inborn error of sulfur amino acid metabolism. CBS operates at the intersection of the transmethylation, transsulfuration, and remethylation pathways. CBS catalyzes the conversion of homocysteine (hcy), an intermediate amino acid derived from the metabolism of dietary methionine, to cystathionine in the first step of the transsulfuration pathway. Cystathionine can be converted to cysteine, thus CBS is critical for the regulation of methionine and cysteine metabolism [1].

Elevated plasma hcy, or hyperhomocysteinemia (hhcy), is categorized by range as moderate (15–30 μM), intermediate (30–100 μM), and severe (above 100 μM). The most common form of severe hhcy is that by CBS deficiency. Patients with CBS deficiency suffer from numerous clinical complications including not only vascular disease, ectopia lentis, osteoporosis, fatty liver but also cognitive dysfunctions such as intellectual disabilities, seizures and psychiatric disorders including personality disorder, anxiety, depression, obsessive-compulsive behavior, and psychotic episodes [1], [2]. Hhcy is also a risk factor for neurodegenerative diseases such as Alzheimer's disease (AD), even if hhcy is moderate [3]. Moreover, elevated hcy level has been associated with neuropsychiatric disorders such as schizophrenia, depression, bipolar disorder, and autism [4], [5], [6], [7]. We showed that hhcys is accompanied by alterations in signaling pathways initiated by receptor tyrosine kinases (RTK), the ERK and Phosphatidylinositol 3-kinase (PI3K)/Akt pathways, and brain-derived neurotrophic factor/TrkB signaling pathway, in brain of CBS deficient mice, a murine model of hhcy [8], [9], [10], [11], [12].

We also demonstrated the relationship between increased DYRK1A level and these signaling pathways in brain of CBS deficient mice [11], [12], [13]. DYRK1A, for dual specificity tyrosine-phosphorylation-regulated kinase 1A, is a serine/threonine kinase that, when overexpressed in brain, contributes to the neurodegeneration, neuronal death, and loss of function observed in multiple neurodegenerative diseases [14]. These results suggest that those could be some of the molecular mechanisms linking hhcy to these neuronal abnormalities. In this line, we observed that enriched environment combined with voluntary exercise enriched environment combined with voluntary exercise restored the altered levels of DYRK1A and brain-derived neurotrophic factor (BDNF), a neurotrophin contributing to hippocampus-dependent forms of learning and memory, in hippocampus of CBS deficient mice [12]. However, no effect was found on plasma hcy level.

Treatment strategies for CBS deficiency generally focus on ways to lower hcy in patients, as this seems to be the best predictor of clinical severity. As the central organ of metabolism, many metabolic diseases originate in the liver. However, clinical manifestations can be extrahepatic. In the case of hhcy, the liver plays a central role in the metabolism of methionine and contributes the high levels of plasma hcy. Recently, we were able to lower plasma hcy levels in mice with moderate and intermediate hhcy using an adenoviral construct designed to restrict the expression of DYRK1A, involved in methionine metabolism (and therefore homocysteine production) [15], to hepatocytes [16], [17]. Interestingly, we found that targeted hepatic expression of DYRK1A abolished the increased DYRK1A level in brain of intermediate hhcy mice, which may be the result of hcy lowering [17].

However, since this adenoviral construct is not suitable for human research, we decided to proceed with an adeno-associated viral (AAV) vector to gain insight about brain molecular readout of DYRK1A overexpression as a hcy lowering therapy. For this, we here used a hepatocyte-specific recombinant AAV serotype 8-mediated Dyrk1A gene transfer (AAV2/8-DYRK1A), AAV serotype 8 having high affinity for hepatocytes without inducing any significant innate immune response. The central nervous system effects of the Dyrk1A gene transfer in hhcy mice were evaluated through the level and activity of proteins related to neuropathological and inflammatory processes.

## 2 Materials and methods

### 2.1 Construction, generation, and production of AVV2/8-DYRK1A

Plasmid pEGFP-DYRK1A containing the 2.6 kb rat Dyrk1a cDNA sequence [18] was used by the Therapeutic Research Institute (Institut de Recherche en Santé de l’Université de Nantes (IRS-UN), Centre de Production de Vecteurs viraux (CPV), Institut National de la santé et de la recherche médicale (INSERM), Unité Mixte de Recherche (UMR) 1089, Nantes, France), to generate and purify AAV2/8-DYRK1A. The expression cassette of this vector consists of the human α1-antitrypsin promoter and one copy of a human apolipoprotein hepatic control region, upstream of the bovine growth hormone polyadenylation signal. Final viral preparations were stored in Dulbecco's Phosphate-Buffered Saline (DPBS) (with Ca2 + and Mg2 +), aliquoted in small volumes (50 µL), and stored at − 80 °C. Particle titer (number of viral genomes) was determined by quantitative PCR poly A. Production and purification processes by this technological platform are consistent with production requirements of clinical grade vectors [19].

### 2.2 Experimental animals

Mice heterozygous for targeted disruption of the *Cbs* gene (Cbs^+/-^) were generously donated by Dr. N. Maeda (Department of Pathology, University of North Carolina, Chapel Hill, NC, USA) [8]. Cbs^+/-^ mice, on a C57BL/6 background, were obtained by mating male Cbs^+/-^ mice with female wild-type C57BL/6 (Cbs^+/+^) mice. DNA isolated from tail biopsies of 4-week-old mice was subjected to genotyping of the targeted *Cbs* allele using polymerase chain reaction (PCR) [8]. Mice were maintained in a controlled environment with unlimited access to food and water on a 12 h light/dark cycle. Mice were fed a standard laboratory diet (CRM, Special Diets Services, Dietex, France Usine). This diet has a protein content of 19%, a methionine content of 2.700 mgKg^-1^, a folic acid content of 4.41 mgKg^-1^, and a vitamin B_12_ content of 0.082 mgKg^-1^. Number of mice and suffering were minimized as possible. Before the experiments and to induce intermediate hhcy the same number of male and female Cbs^+/-^ mice of 3 months of age were maintained for three months on the standard diet supplemented with 0.5% L-methionine (Sigma-Aldrich, France) in drinking water.

We were not powering for rigourously testing sex differences, but preliminary analysis showed no differences for plasma hcy level. Therefore, here we combined data from male and female. Cbs^+/-^ mice and mice with intermediate hhcy (Cbs^+/-^ mice having the methionine enriched diet) were divided into two groups for the last month, with one group (hhcy/AAV) receiving injection in the retro-orbital sinus with the AAV2/8-DYRK1A to have 2 × 10^12^ viral particles/kg body weight and the second group receiving an equivalent dose of saline buffer. Control mice (CTL), healthy control Cbs^+/+^ mice also received injection in the retro-orbital sinus with the AAV2/8-DYRK1A or an equivalent dose of saline buffer and were used as references to monitor hyperhomocysteinemic development.

All procedures were carried out in accordance with the ethical standards of French and European regulations (European Communities Council Directive, 86/609/EEC). Official authorization from the French Ministry of Agriculture was granted to perform research and experiments on animals (authorization number 75–369) and the experimental protocol was approved by the institutional animal care and use committee of the Paris Diderot University (CEEA40).

### 2.3 Preparation of serum samples, tissue collection, and plasma assays

Mice were anesthetized by Ketamine/Xylazine intraperitoneal injection, and blood samples were obtained by retro-orbital sinus sampling with heparinized capillaries, collected into tubes containing a 1/10 vol of 3.8% sodium citrate, and immediately placed on ice. Plasma was isolated by centrifugation at 2500 × g for 15 min at 4 °C. Livers and brains were harvested, snap-frozen, and stored at −80 °C until use. Plasma total hcy, defined as the total concentration of hcy after quantitative reductive cleavage of all disulfide bonds, was assayed using the fluorimetric high-performance liquid chromatography (HPLC) method as previously described [20]. The DYRK1A levels were assessed by a solid phase immobilized epitope immunoassay, as described [21].

### 2.4 Protein extraction and analysis

Total protein samples were prepared by homogenizing liver and brain in 500 µL phosphate-buffered saline with a cocktail of proteases inhibitors. Cytoplasmic and nuclear proteins were obtained by using the NE-PER Nuclear Protein Extraction Kit (Thermo Scientific). Protein concentrations were detected with the Bio-Rad Protein Assay reagent (Bio-Rad). To assess the relative amount of proteins, we used a slot blot method after testing the specificity of antibodies by western blotting. Protein preparations were blotted on Hybond-C Extra membrane (GE Healthcare Europe GmbH) using Bio-Dot SF Microfiltration Apparatus (Bio-Rad). After transfer, membranes were saturated by incubation in 10% w/v nonfat milk powder or 5% w/v BSA in Tris-saline buffer (1.5 mM Tris base, pH 8; 5 mM NaCl; 0.1% Tween-20), and incubated overnight at 4 °C with an antibody directed against Akt1/ 2/3 (1/1000; Santa Cruz Biotechnology, Tebu, France), Phospho-Akt1/2/3 (Ser 473; 1/1000; Santa Cruz Biotechnology, Tebu, France), p44/42 MAPK (ERK1/2)(137F5) (1/2000; Cell Signaling, Ozyme, France), Phospho-p44/42 MAPK (ERK1/2) (Thr202/Tyr204) (1/2000; Cell Signaling, Ozyme, France), IkBα (1/1000, Cell Signaling Technology), P- IkBα (Ser32) (1/1000, Cell Signaling Technology), Calpain 2 Domain I (1/5000, abcam, France), Calpastatin (1/1000, Cell Signaling Technology), DYRK1A (1/500, Abnova corporation, Tebu, France), NFkB p65 (1/2000, abcam, France). Binding of the primary antibody was detected by incubation with horseradish peroxidase (HRP)-conjugated secondary antibody using the Western Blotting Luminol Reagent (Santa Cruz Biotechnology, Tebu, France). Ponceau-S coloration (Sigma-Aldrich, France) was used as an internal control. Digitized images of the immunoblots obtained using LAS-3000 imaging system (Fuji Photo Film Co., Ltd.) were used for densitometric measurements with an image analyzer (UnScan It software, Silk Scientific Inc.).

### 2.5 Enzyme activity assays

CBS activity assay was performed on 300 µg of protein extracts as described previously [22]. Proteins were incubated for 1 h at 37 °C with 1 mM DL-propargylglycine, 0.2 mM pyridoxal 5’-phosphate, 10 mM L-serine, 10 mM DL-Hcy, and 0.8 mM S-(5’-adenosyl)-L-methionine, using 5,5’-dithiobis-(2-nitrobenzoic acid) based-assay. The reaction was performed at 37 °C by measuring the absorbance at 412 nm over 10 min, using a spectrophotometer (Lambda XLS, PerkinElmer). Calpain activity was measured using the fluorogenic peptide N-Succinyl-Leu-Tyr-7-Amido-4-Methylcoumarin as described by Kohli et al. [23]. Briefly, 60 µg of brain extract in a final volume of 40 µL was added to 160 µL of 50 µM N-Succinyl-Leu-Tyr-7-Amido-4-Methylcoumarin dissolved in dimethyl sulfoxide and Tris buffer (100 mM Tris-HCl, 145 mM NaCl at pH 7.3). Proteolysis of the substrate was monitored for 21 min at room temperature with a FlexStation3 multi-mode microplate reader (excitation: 380 nm, emission: 460 nm; Molecular Devices) either in presence of 10 mM Ca^2+^ or 10 mM EGTA to determine calcium-independent activity, thus excluding cathepsin activity. Protein phosphatase 2 A (PP2A) activity assay was performed using the PP2A immunoprecipitation phosphatase assay kit (Millipore) using 5 mg of protein extract as described by the manufacturer.

### 2.6 BDNF protein analysis

BDNF protein levels were measured in the brain using the BDNF EMax Immunoassay (ELISA E-Max, Promega, Madison, WI, USA). Protein preparations were incubated on a 96-well polystyrene ELISA plate previously coated with anti-BDNF monoclonal antibody. A standard curve was generated from serial dilutions of a human recombinant BDNF solution at 1 μg/mL. The captured neurotrophin was bound by a second specific anti-human BDNF polyclonal antibody, which was detected using a species-specific antibody conjugated to horseradish peroxidase (HRP). After removal of unbound conjugates, bound enzyme activity was assessed by chromogenic substrate for measurement at 450 nm by a microplate reader (Flex Station3, Molecular Device). All assays were performed in duplicate.

### 2.7 RNA extraction, cDNA synthesis, and real-time PCR using SYBR-Green chemistry

Total RNA was isolated from the brain and liver using RNeasy Lipid kit (Qiagen). The concentration of RNA samples was ascertained by measuring optical density at 260 nm. The quality of RNA was verified by optical density absorption ratio OD 260 nm/OD 280 nm. To remove residual DNA contamination, the RNA samples were treated with RNAse-free DNAse (Qiagen) and purified with an RNeasy mini column (Qiagen). For each sample, 4 µg of total RNA from each sample was reverse transcribed with 200 U of M-MLV Reverse Transcriptase (Invitrogen, Life Technologies) using random hexamer primers. Real time quantitative PCR amplification reactions were carried out in a LightCycler 480 detection system (Roche) using the LightCycler FastStart DNA Master plus SYBR Green I kit (Roche). Primer sequences are ^5’^TCAGTCTTCAGGCACCACCT^3’^ (sense primer) and ^5’^TGTTACTCGTTCCCGAGGAT^3’^ (antisense primer) for DYRK1A. For each reaction, 40 ng of reverse transcribed RNA was used as template. All reactions were carried out in duplicate with a no template control. The PCR conditions were: 95 °C for 5 min, followed by 45 cycles of 95 °C for 10 s, 60 °C for 10 s and 72 °C for 10 s. The mRNA transcript level was normalized against the mean of two genes: H1a and rpL19. Primer sequences are ^5’^AGAAGAACAACAGCCGCATC3’ (sense primer) and ^5’^TGCACCAGTGTGCCTTTATT^3’^ (antisense primer) for H1a, and ^5’^GGGCAGGCATATGGGCATA^3’^ (sense primer) and ^5’^GGCGGTCAATCTTCTTGGATT^3’^ (antisense primer) for rpL19. To compare the target gene level, relative quantification was performed as outlined in Pfaffl et al. [24].

### 2.8 Data analysis

For multiple pairwise comparisons between genotypes and treatments, statistical analysis was done with two-way ANOVA followed by Fisher's post hoc test using Statview software. The results are expressed as mean ± SEM (standard error of the mean). Data were considered significant when p < 0.05. Correlations were determined by using Spearman's rank correlation, as data were not normally distributed according to Shapiro-Wilk test.

## 3 Results

### 3.1 Effect of AAV2/8-DYRK1A gene transfer on plasma hcy level in mice with hhcy

**Fig. 1:**
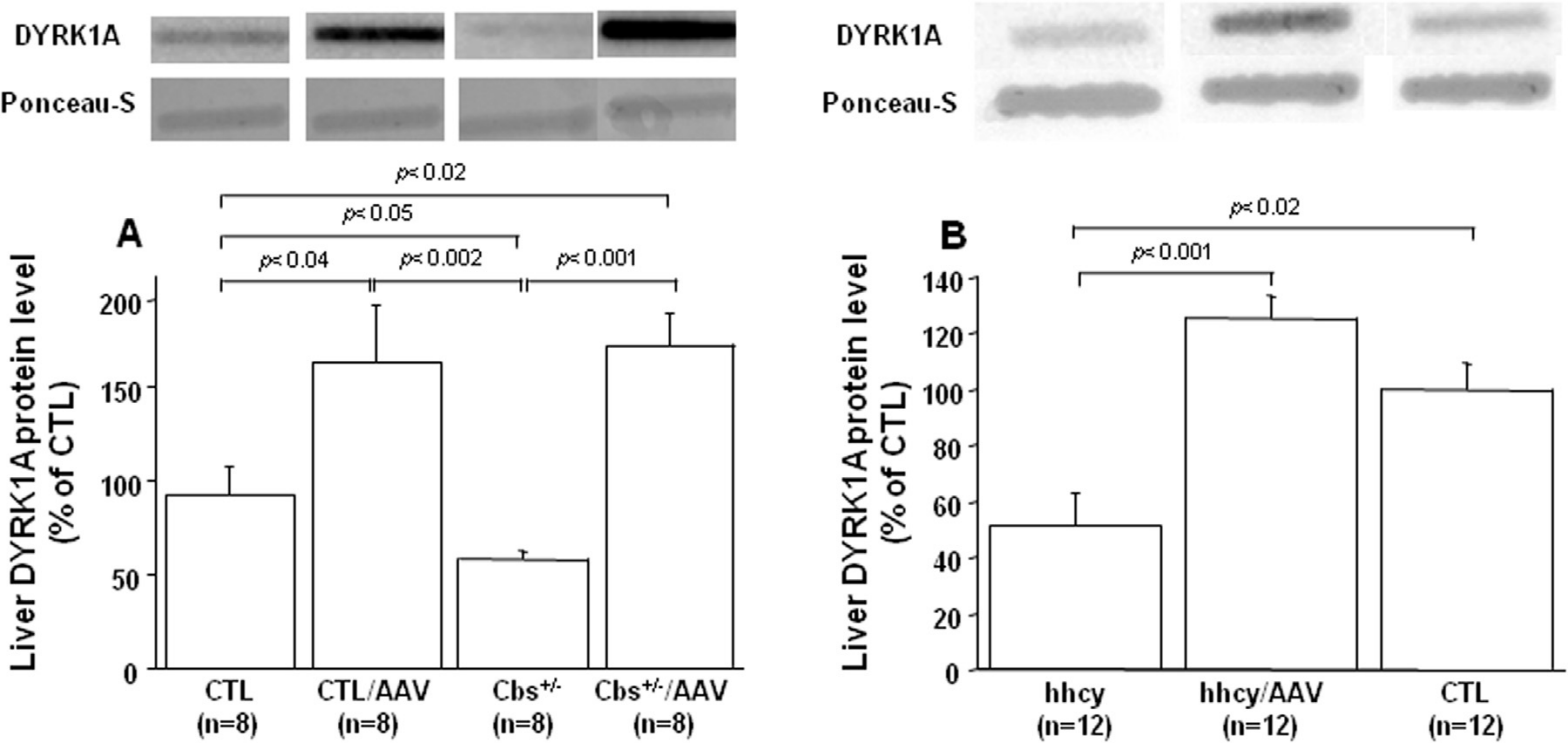
**Effect of AVV2/8-DYRK1A gene transfer on hepatic DYRK1A level.** A) Liver DYRK1A protein level in control (CTL) mice, Cbs^+/-^ mice, and B) Cbs^+/-^ mice with intermediate hhcy (hhcy) with or without AAV2/8-DYRK1A treatment (AAV). Liver DYRK1A was determined by slot blotting, and values were obtained by normalization of images from DYRK1A to total proteins colored with Ponceau-S. Data were normalized to the mean of control (CTL) mice. The results are represented as means ± SEM. n = number of mice.

**Fig. 2:**
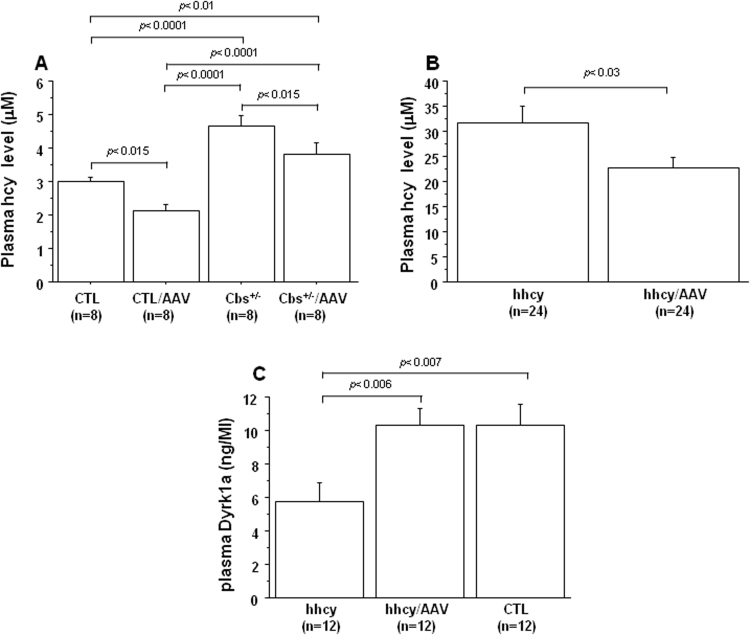
**Effect of AVV2/8-DYRK1A gene transfer on plasma hcy and DYRK1A level.** A) Plasma hcy level in A) control (CTL) mice, Cbs^+/-^ mice and B) Cbs^+/-^ mice with intermediate hhcy (hhcy) with or without AAV2/8-DYRK1A treatment (AAV). C) Plasma DYRK1A level in mice with intermediate hhcy with or without AAV2/8-DYRK1A treatment (AAV), determined by ELISA. The results are represented as means ± SEM. n = number of mice.

Similar to previous reports [16], [17], [25], mean DYRK1A protein level was decreased in the liver of Cbs^+/-^ mice (Fig. 1A) and mice with intermediate hhcy (Fig. 1B) when compared with control mice. However, after injection of AAV2/8-DYRK1A, we observed an increase in DYRK1A protein level when compared with mice without treatment. The mRNA expression of DYRK1A was also examined by Q-PCR and, as previously described [25], even if the difference is not significant, the mRNA expression was increased in liver of mice with intermediate hhcy (hhcy), when compared with control (CTL) mice (Table 1). Moreover, the increased expression of protein DYRK1A was confirmed by Q-PCR analysis in liver of mice with intermediate hhcy after injection of AAV2/8-DYRK1A (hhcy/AAV) (Table 1). We therefore determined the effect of AAV2/8-DYRK1A on plasma hcy levels, and found that control mice, Cbs^+/-^ mice (Fig. 2A) and mice with hhcy (Fig. 2B) had significantly lower plasma hcy level compared to mice without treatment. We interestingly observed an increased level of plasma DYRK1A in mice with intermediate hhcy after AAV2/8-DYRK1A injection (Fig. 2C).

**Table 1:** Relative liver and brain mRNA expression of DYRK1A based upon Q-PCR data obtained from control (CTL), Cbs^+/-^ mice with intermediate hyperhomocysteinemia (hhcy) with (hhcy/AAV) or without (hhcy) AAV2/8-DYRK1A treatment.

Genotype	Relative expression of DYRK1A mRNA in liver	Relative expression of DYRK1A mRNA in brain
CTL	100 ± 38 (n = 4)	100 ± 9 (n = 4)
hhcy	165 ± 20 (n = 4)	86 ± 2 (n = 4)
hhcy/AAV	540 ± 159^**, $^ (n = 4)	87 ± 9 (n = 4)

Data were normalized to the mean of control (CTL) mice. Data are presented as mean ± SEM. n = number of mice. * p < 0.01 (compared to CTL mice); $ p < 0.02 (compared to hhcy mice).

### 3.2 Effect of AAV2/8-DYRK1A gene transfer on DYRK1A protein level and CBS activity in brain of mice with intermediate hhcy

**Fig. 3:**
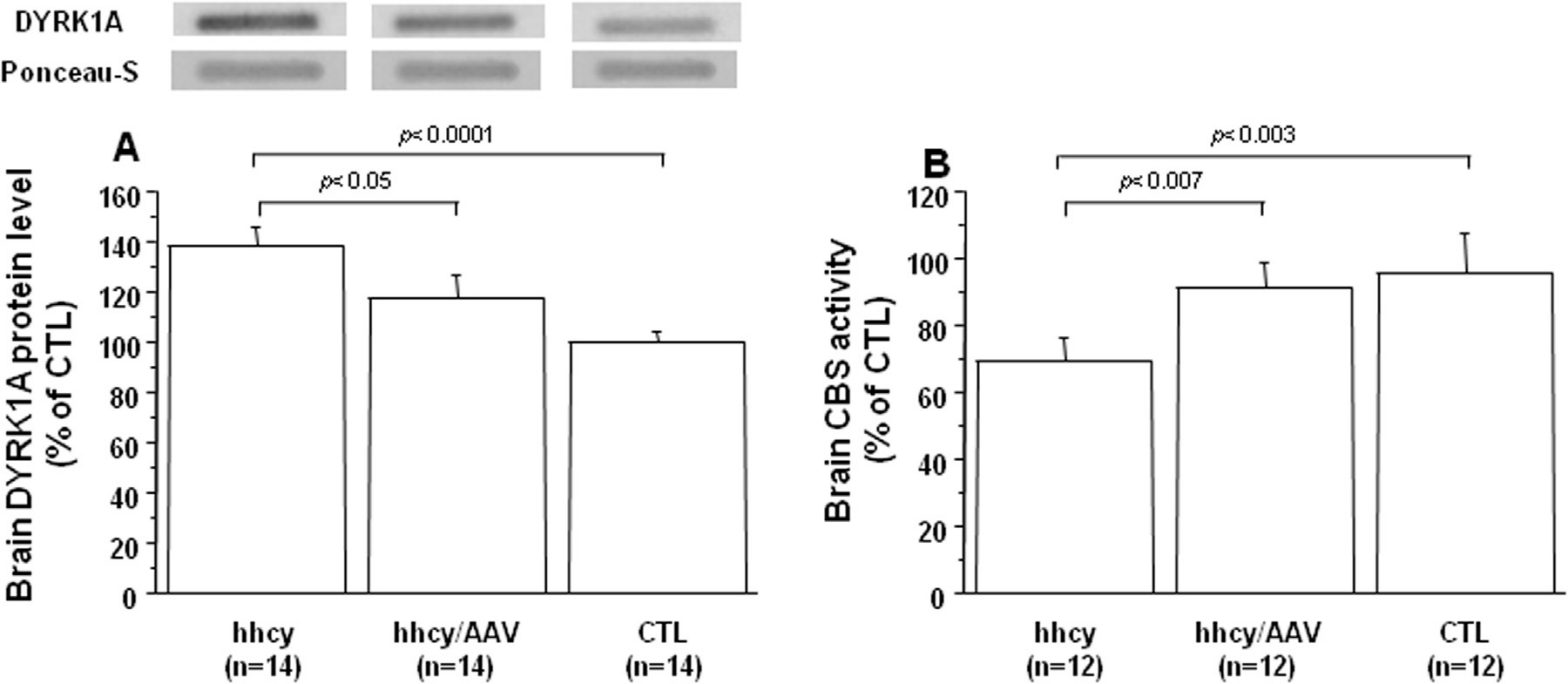
**Effect of AVV2/8-DYRK1A gene transfer on brain DYRK1A and CBS activity level.** A) DYRK1A protein level, and B) CBS activity in brain of Cbs^+/-^ mice with intermediate hhcy with (hhcy/AAV) or without (hhcy) AAV2/8-DYRK1A treatment, and control (CTL) mice. Brain DYRK1A was determined by slot blotting, and values were obtained by normalization of images from DYRK1A to total proteins colored with Ponceau-S. Data were normalized to the mean of CTL mice. The results are represented as means ± SEM. n = number of mice.

Similar to our previous reports [12], [13], mean DYRK1A protein level was increased in brain of mice with intermediate hhcy when compared with control (CTL) mice (Fig. 3A). However, the mRNA expression of DYRK1A was not changed in brain of mice with intermediate hhcy (hhcy) when compared with control (CTL) mice (Table 1), as previously described [13]. Furthermore, after injection of AAV2/8-DYRK1A, we observed a decrease in DYRK1A protein level when compared with mice with hhcy and without treatment (hhcy) (Fig. 3A), but without effect on mRNA expression of DYRK1A (Table 1). Taken together, the results on brain DYRK1A protein and mRNA levels also emphasize the liver specific expression after AAV2/8-DYRK1A injection. Moreover, treatment can also increase the CBS activity in brain of mice with hhcy (Fig. 3B).

### 3.3 Effect of AAV2/8-DYRK1A gene transfer on pathways initiated by RTK in brain of mice with intermediate hhcy

**Fig. 4:**
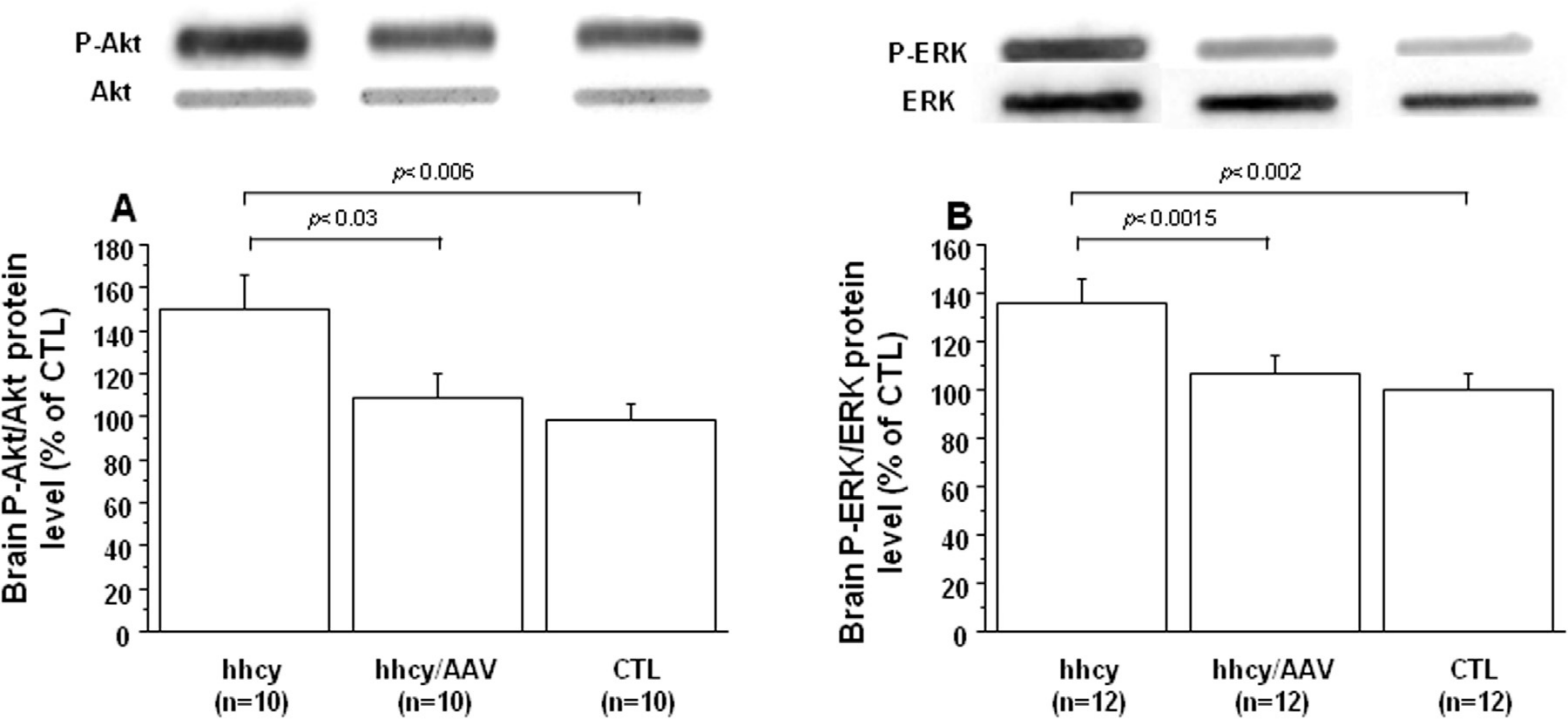
**Effect of AVV2/8-DYRK1A gene transfer on brain RTK activation.** A) Akt and B) ERK activation in brain of Cbs^+/-^ mice with intermediate hyperhomocysteinemia (hhcy) with (hhcy/AAV) or without (hhcy) AAV2/8-DYRK1A treatment, and control (CTL) mice. Proteins were subjected to slot blot analysis using antibodies specific to P-akt or P-ERK. After stripping, the membranes were reprobed with anti Akt or anti ERK antibody for the control. Relative protein expression was determined by normalization of the density of images from P-Akt or P-ERK with that of Akt or ERK of the same blot. Data were normalized to the mean of CTL mice. The results are represented as means ± SEM. n = number of mice.

PI3K can become activated by at least three independent pathways, all of which start with binding of ligand to RTK. Activation of Ras can coordinate activation of PI3K and Raf/Mek/Erk. Therefore, Akt and ERK activation was analyzed using phospho-specific antibodies. Phospho-Akt (P-Akt) and phospho-ERK (P-ERK) levels were increased in brain of mice with intermediate hhcy compared to control mice, as previously described [11] (Fig. 4). However, after injection of AAV2/8-DYRK1A, we observed reduced Phospho-Akt and phospho-ERK levels when compared with mice with intermediate hhcy and without treatment (Fig. 4).

### 3.4 Effect of AAV2/8-DYRK1A gene transfer on BDNF level in brain of mice with intermediate hhcy

**Fig. 5:**
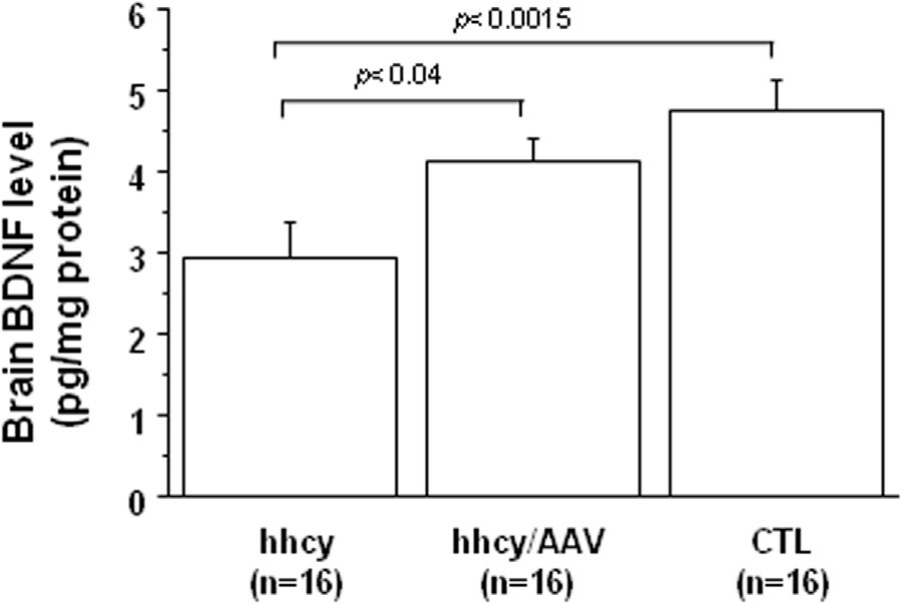
**Effect of AVV2/8-DYRK1A gene transfer on brain BDNF level.** BDNF levels were measured by ELISA in brain of Cbs^+/-^ mice with intermediate hyperhomocysteinemia (hhcy) with (hhcy/AAV) or without (hhcy) AAV2/8-DYRK1A treatment, and control (CTL) mice. The results are represented as means ± SEM. n = number of mice.

BDNF levels were decreased in brain of mice with intermediate hhcy compared to control mice, as previously described [12] (Fig. 5). Following AAV2/8-DYRK1A injection, BDNF protein levels significantly increased when compared with mice with intermediate hhcy and without treatment (Fig. 5).

### 3.5 Effect of AAV2/8-DYRK1A gene transfer on NFkB pathway in brain of mice with intermediate hhcy

**Fig. 6:**
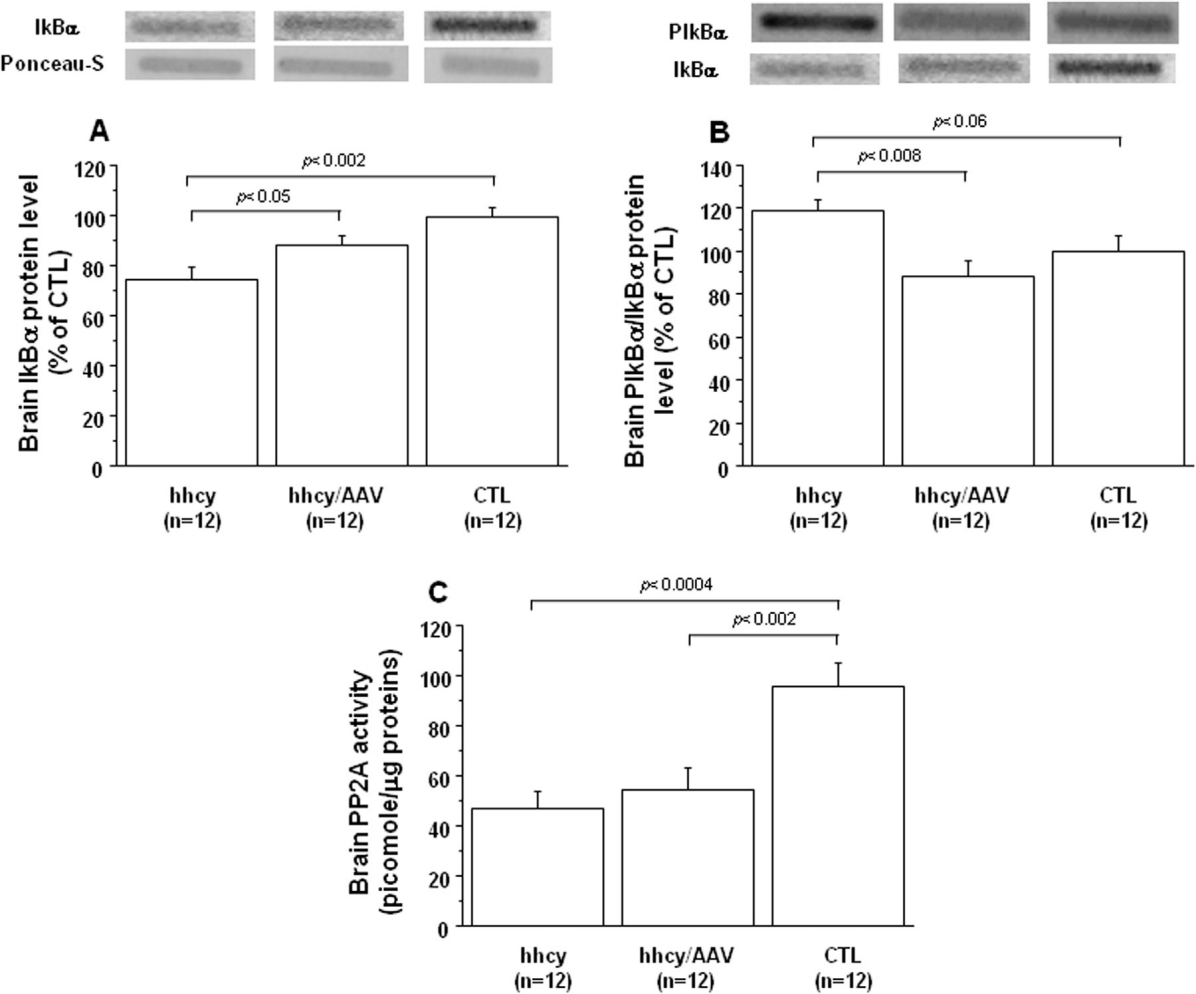
**Effect of AVV2/8-DYRK1A gene transfer on brain IkBα degradation.** A) IkBα protein level, B) Ser32-phosphorylated IkBα and C) PP2A activity in brain of Cbs^+/-^ mice with intermediate hyperhomocysteinemia (hhcy) with (hhcy/AAV) or without (hhcy) AAV2/8-DYRK1A treatment, and control (CTL) mice. Proteins were subjected to slot blot analysis using antibodies specific to P-IkBα. After stripping, the membranes were reprobed with anti IkBα antibody. Relative protein expression was determined by normalization of the density of images from P- IkBα with that of IkBα of the same blot. Values of IkBα were obtained by normalization of images from IkBα to total proteins colored with Ponceau-S. PP2A activity was determined by immunoprecipitation phosphatase assay as described in material and methods Data were normalized to the mean of CTL mice. The results are represented as means ± SEM. n = number of mice.

To determine whether the NF-kB pathway is activated in brain of mice with intermediate hhcy, we studied the level of IkBα and found a decrease compared to control (CTL) mice (Fig. 6A). The first possible way for IκBα degradation implicates the balance of IKKβ /PP2A. We quantified the ratio of phospho-IκBα/IκBα (Fig. 6B) and PP2A activity (Fig. 6C) and found an increase and a decrease respectively compared to control (CTL) mice. Following AAV2/8-DYRK1A injection, IkBα protein levels significantly increased when compared with mice with intermediate hhcy and without treatment, with a decrease of phospho-IκBα/IκBα ratio but no difference for PP2A activity (Fig. 6).

**Fig. 7:**
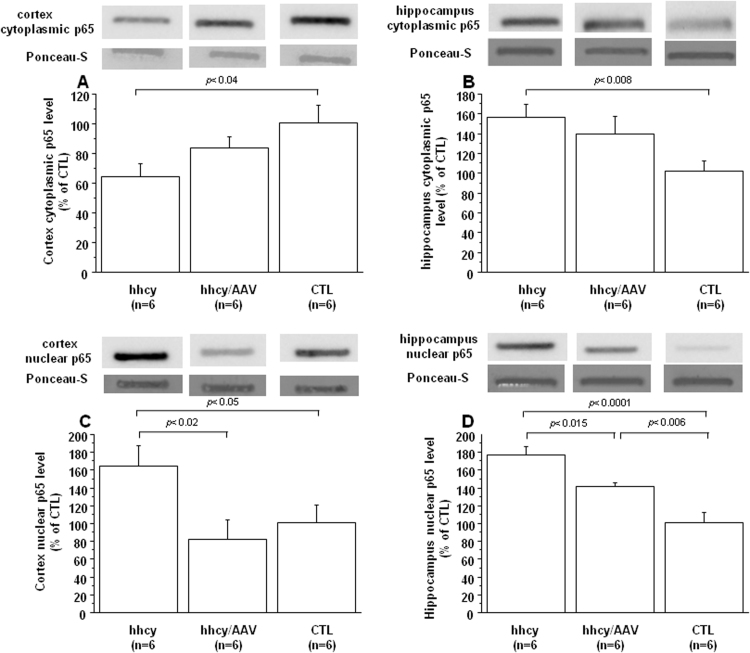
**Effect of AVV2/8-DYRK1A gene transfer on brain p65 localization.** Cytoplasmic p65 levels in A) cortex and B) hippocampus, and nuclear p65 levels in C) cortex and D) hippocampus in brain of Cbs^+/-^ mice with intermediate hyperhomocysteinemia (hhcy) with (hhcy/AAV) or without (hhcy) AAV2/8-DYRK1A treatment, and control (CTL) mice. Values were obtained by normalization of images from nuclear and cytoplasmic p65 to total proteins colored with Ponceau-S. Data were normalized to the mean of CTL mice. The results are represented as means ± SEM. n = number of mice.

IkB inhibits NFkB by sequestering the NFkB p65 subunit in the cytoplasm. Into the nucleus, p65 provides the activation of the transcription factor complex. We therefore analyzed the subcellular p65 localization in brain of mice and found a decrease in the cytoplasmic cortex fraction (Fig. 7A) but an increase in the cytoplasmic hippocampus fraction (Fig. 7B) of mice with intermediate hhcy compared to control (CTL) mice. The nuclear p65 fraction was increased in cortex and hippocampus of mice with intermediate hhcy (Figs. 7C and 7D). However, after injection of AAV2/8-DYRK1A, we observed reduced nuclear p65 levels both in cortex and hippocampus when compared with mice with intermediate hhcy and without treatment (Figs. 7C and 7D).

**Fig. 8:**
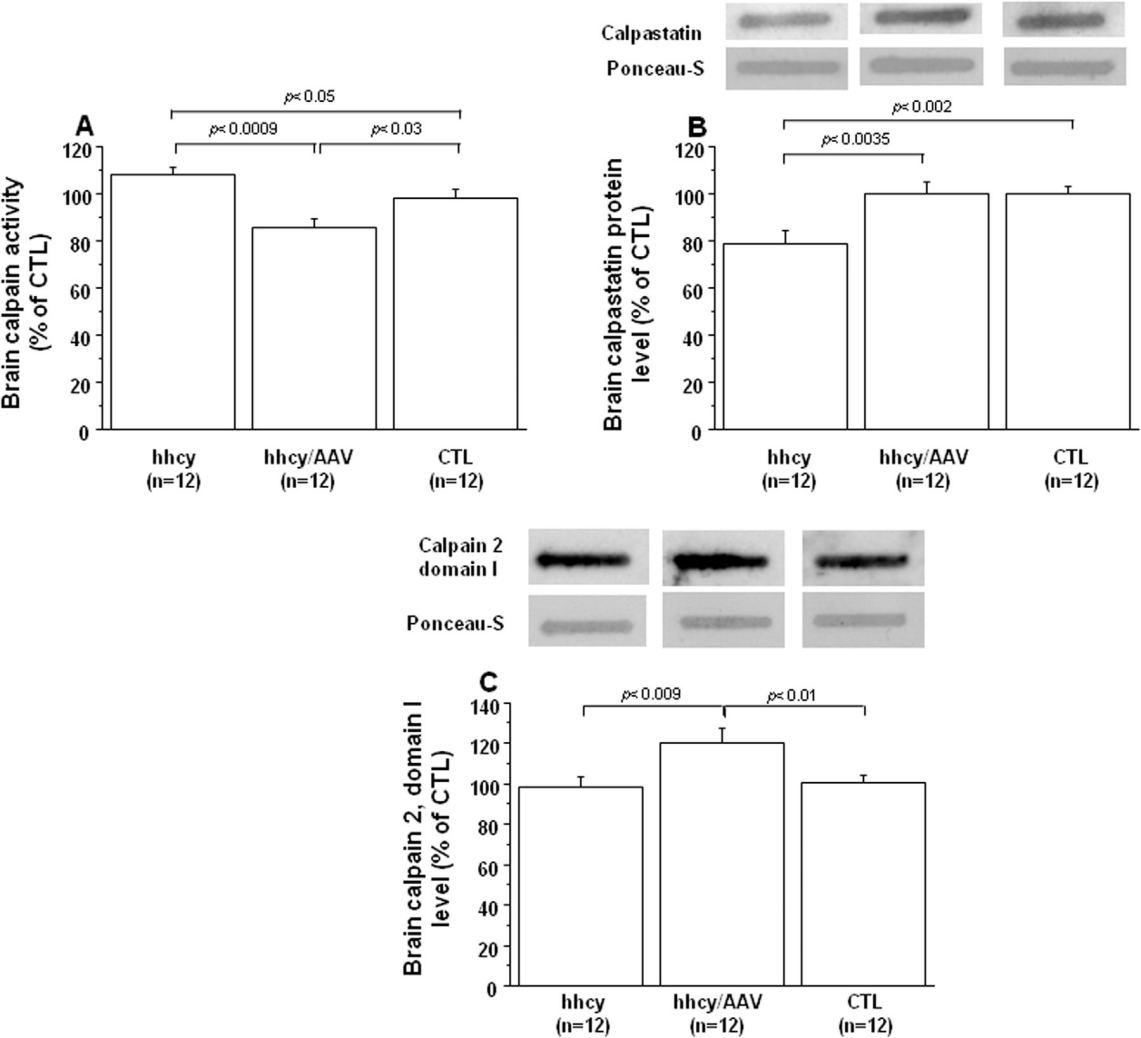
**Effect of AVV2/8-DYRK1A gene transfer on brain calpain activity.** A) Calpain activity, B) calpastatin and C) calpain 2 domain I protein levels in brain of Cbs^+/-^ mice with intermediate hyperhomocysteinemia (hhcy) with (hhcy/AAV) or without (hhcy) AAV2/8-DYRK1A treatment, and control (CTL) mice. Calpain activity was determined by measuring the proteolysis of the substrate for 21 min. Calpastatin and calpain 2 domain I protein values were obtained by normalization of images from calpastatin and calpain 2 domain I to total proteins marked with Ponceau-S. Data were normalized to the mean of CTL mice. The results are represented as means ± SEM. n = number of mice.

The second possible way for IκBα degradation implicates the balance of calpain/calpastatin. We found an increased calpain activity (Fig. 8A), with a decreased calpastatin level (Fig. 8B), in brain of mice with intermediate hhcy. Following AAV2/8-DYRK1A injection, calpain activity significantly decreased (Fig. 8A) when compared with mice with intermediate hhcy and without treatment, with an increase of calpastatin level (Fig. 8B). We also analyzed the protein level of calpain 1 and calpain 2, the two major calpain isoforms in the brain. Calpain are calcium-dependent proteases, with a large subunit containing domains I-IV. No difference was found for domain I, the propeptide domain, of calpain 1, nor for domain IV, a Ca2 + -binding domain, of calpain 1 and 2 (data not shown). However, an increase of calpain 2 domain I protein level was found in brain of mice with intermediate hhcy after AAV2/8-DYRK1A injection compared to control and mice with intermediate hhcy (Fig. 8C).

## 4 Discussion

High levels of plasma hcy have been linked to the development of neurological disorders associated with neuronal loss such as AD, vascular dementia or mild cognitive impairment [26], [27]. We found a positive correlation between plasma hcy level and brain DYRK1A protein level (r = 0.278, p < 0.05), as well as a negative correlation between brain CBS activity and plasma hcy (r = -0.409, p < 0.03). We have previously produced moderate and intermediate hyperhomocysteinaemia in mice in order to monitor the levels of hepatic CBS activity and found that not only methionine enriched diet increased plasma hcy levels but also decreased hepatic CBS activity in Cbs^+/-^ mice [28]. All together, these results underline the effect of intermediate hhcy on brain protein DYRK1A level and CBS activity. We showed that increasing hepatic expression of DYRK1A in mice with intermediate hhcy results in plasma hcy lowering. The decrease brain DYRK1A level and the increase brain CBS activity would result from plasma hcy lowering [17].

The study on transgenic mice overexpressing DYRK1A [11] established a relationship between DYRK1A protein and RTK-mediated signaling pathways such as MAPK/ERK and PI3K/Akt pathways as well as the BDNF-dependent TrkB pathway in brain of mice [29]. We confirmed the activation of ERK and Akt, as well as the decreased BDNF content in brain of mice with hhcy [11], [12], [30]. Positive correlations were found between brain DYRK1A protein level and ERK and Akt activation (r = 0.601, p < 0.0001). We previously found decreased activation of ERK and Akt pathways associated with decreased DYRK1A protein level in liver of mice with hhcy [17], [25], [31]. The observed negative correlation between plasma hcy level and brain BDNF level (r = -0.444, p < 0.004) suggest a negative effect of hhcy on BDNF/TrkB signaling pathway, as previously described [12]. Taken together, our results emphasize the role of DYRK1A and hhcy on RTK and TrkB pathway regulation.

It has been shown in primary cortical neurons that glutamate-induced triggering of the ionotropic N-methyl-D-aspartate (NMDA) receptor (NMDAR) was required for the enhanced and persistent Pi3k/Akt-dependent NF-kappa B activation by the 75 kDa tumor necrosis factor receptor (TNFR2), indicating a positive cooperation of TNF and neurotransmitter-induced signal pathways [32]. We previously demonstrated that both mGluRs, NMDAR and non-NMDAR, cooperatively mediate hcy-induced ERK activation, indicating complex cellular mechanisms that render the hippocampus sensitive to hcy [10]. Expression of serine / threonine phosphatase PP2A, a phosphatase, that supress IkB phosphorylation by IKK, is inhibited by the PI3K/Akt pathway in primary cultures of neurons [33], [34]. PP2A may also be involved in the inactivation of MEK and ERK [35]. Previous study demonstrated that impaired hcy metabolism and reduced PP2A methylation levels could trigger the accumulation of phosphorylation of tau and APP in the brain, a process that may favor neurofibrillary tangle formation and amyloidogenesis [36]. We therefore analyzed the PP2A activity in brain of mice with intermediate hhcy and found that, commensurate with the decreased PP2A activation, they present decreased level of IkBα with an increased level of its phosphorylation. In addition, a negative and a positive correlation were found between plasma hcy level and IkBα protein level (r = -0.415, p < 0.03) and phosphorylation of IkBα (r = 0.404, p < 0.04) respectively. In absence of stimuli, NFkB is sequestered in the cytoplasm by the protein IkBα. Following a stimulus, IkBα is phosphorylated, resulting in the dissociation of the complex NFkB/IkBα and the translocation of NFkB in the nucleus [37]. We therefore analyzed the p65 level and found an increase in nuclear fraction in cortex and hippocampus of mice with intermediate hhcy. This increase was found to be associated with a decrease cytoplasmic p65 level in cortex. An increase cytoplasmic p65 level was found in hippocampus of mice with intermediate hhcy, as previously described in rats after chronic hcy administration [38], with a positive correlation between cytoplasmic and nuclear p65 levels in mice hippocampus (r = 0.653, p < 0.02). The increased nuclear p65 level emphasizes the activation of NFkB pathway in brain of mice with intermediate hhcy.

In addition to the link between PI3K/Akt pathway and PP2A activity on IkBα stability, Ras (a small GTP-binding protein) activated by RTK, enhances NFkB transcriptional activity through a Raf-independent mechanism [39], [40]. We previously demonstrated that hcy-mediated IkBα enhanced proteolysis occurred via calcium-dependent calpains, which was supported by an increased level of calpain activity and a reduced expression of calpastatin in liver of mice with hhcy [41]. Mice with intermediate hhcy also showed increased brain calpain activity, with a decreased calpastatin level, suggesting the involvement of the calpain-calpastatin system in reducing the level of IkBα in response to intermediate hhcy. Taken together, our results emphasize the deleterious effect of hhcy on NFkB pathway, due not only by RTK activation but also in parallel by NMDAR activation and calpain inducing effect.

Low plasma BDNF level has been associated with high plasma hcy level in drug naïve, first episode schizophrenia patients compared to healthy control group, which may play an important role in the neurodevelopmental process and clinical manifestation of schizophrenia [42]. Interestingly, there was a notable increase in plasma hcy level and significant decrease in serum BDNF level in amnestic mild cognitive impairment patients that converts to AD patients, especially in those with the APOE ε4 allele [43]. Cognitive impairment is also closely related to inflammatory responses, which is highly regulated by many factors, including NFkB pathway [44], [45]. Exacerbated activation of calpain has been implicated as a major component in the signaling cascade that leads to β-amyloid (Aβ) production and tau hyperphosphorylation in AD, leading to a hypothesis that selective calpain inhibitors are potential therapeutic strategies for AD [46], [47]. ERK kinases can phosphorylate tau and induce tau-dependent and tau-independent pathogenic effects, including synaptic plasticity defects and cognitive impairment [48]. Therefore, we analyzed the effect of this selective hcy lowering therapy upon molecular mechanisms implicated not only in neuropathological but also in inflammatory processes. After treatment, we found an increased BDNF level compared to mice with intermediate hhcy and without treatment. Previous study also demonstrated a positive effect of hcy lowering on BDNF level in hippocampus and short- and long-term memories in rats [30]. ERK, Akt and IkBα phosphorylation were found to be decreased. Commensurate with the decreased level of IkBα phosphorylation, we found increased IkBα level and decreased level of nuclear p65 after AAV2/8-DYRK1A gene transfer. However, no effect was found for PP2A activity in brain of mice with intermediate hhcy after treatment. The increased IκBα protein level is associated with a reduction of calpain activity, which is associated with increased calpastatin level in brain of mice with intermediate hhcy after AAV2/8-DYRK1A gene transfer. The increase of calpain 2 domain I suggests a positive feedback to maintain its homeostasis.

## 5 Conclusion

Taken together, our results demonstrate that targeting hepatic DYRK1A expression can correct plasma DYRK1A and hcy level in mice with intermediate hhcy. The consequences of plasma hcy lowering therapy are restoration of brain DYRK1A levels, BDNF levels, ERK, Akt and IkBα phosphorylation levels as well as calpain-calpastatin system and CBS activity, therefore, thus protecting from neuropathological and inflammatory processes. The inhibition of calpain activity and RTK-mediated signaling pathways after AAV2/8-DYRK1A gene transfer suggest a potential therapeutic effect of hcy lowering in the treatment of tauopathies and neurodegenerative states.
